# Short-Term Associations between Air Pollution Concentrations and Respiratory Health—Comparing Primary Health Care Visits, Hospital Admissions, and Emergency Department Visits in a Multi-Municipality Study

**DOI:** 10.3390/ijerph14060587

**Published:** 2017-05-31

**Authors:** Tahir Taj, Ebba Malmqvist, Emilie Stroh, Daniel Oudin Åström, Kristina Jakobsson, Anna Oudin

**Affiliations:** 1 Department of Occupational and Environmental Medicine, Lund University, SE-22100 Lund, Sweden; ebba.malmqvist@med.lu.se (E.M.); emilie.stroh@med.lu.se (E.S.); kristina.jakobsson@med.lu.se (K.J.); anna.oudin@umu.se (A.O.); 2 Center for Primary Health Care Research, Department of Clinical Science, Malmö, Lund University, SE-205 02 Malmö, Sweden; daniel.oudin_astrom@med.lu.se; 3 Department of Occupational and Environmental Medicine, Umeå University, Umeå, SE-22100 Lund, Sweden

**Keywords:** air pollution, primary health care, respiratory health, hospital admissions and ER visits, case crossover

## Abstract

Acute effects of air pollution on respiratory health have traditionally been investigated with data on inpatient admissions, emergency room visits, and mortality. In this study, we aim to describe the total acute effects of air pollution on health care use for respiratory symptoms (ICD10-J00-J99). This will be done by investigating primary health care (PHC) visits, inpatient admissions, and emergency room visits together in five municipalities in southern Sweden, using a case-crossover design. Between 2005 and 2010, there were 81,019 visits to primary health care, 38,217 emergency room visits, and 25,271 inpatient admissions for respiratory symptoms in the study area. There was a 1.85% increase (95% CI: 0.52 to 3.20) in the number of primary health care visits associated with a 10 µg/m^3^ increase in nitrogen dioxide (NO_2_) levels in Malmö, but not in the other municipalities. Air pollution levels were generally not associated with emergency room visits or inpatient admissions, with one exception (in Helsingborg there was a 2.52% increase in emergency room visits for respiratory symptoms associated with a 10 µg/m^3^ increase in PM_10_). In conclusion, the results give weak support for short-term effects of air pollution on health care use associated with respiratory health symptoms in the study area.

## 1. Introduction

Respiratory diseases are one of the leading causes of both morbidity and mortality worldwide [1,2]. Lung infections, chronic obstructive pulmonary diseases (COPD), asthma, and lung cancer are main contributors of both respiratory-related mortality and morbidity [3]. In the European Union (EU), respiratory diseases are responsible for 7% of the total number of hospital admissions [4], and it has been estimated that one in eight deaths is directly attributed to respiratory illness [4]. By 2030, the WHO estimates that respiratory diseases will be the major cause of impaired quality of life and death in the EU and that it will require substantial economic resources to both prevent and treat patients [5]. It is therefore important to identify risk factors for respiratory diseases in order to identify preventive measures.

Several epidemiological studies have found associations between daily concentrations of ambient air pollution, such as particulate matter (PM), ozone (O_3_), and nitrogen dioxide (NO_2_), and daily mortality and morbidity outcomes due to respiratory diseases [6,7,8,9,10,11]. An increased number of inpatient admissions and emergency visits have been reported after a short-term increase in pollutants as well as long-term exposure [6,12,13]. Interestingly, these adverse effects have also been shown in low-level air pollution settings [8,14,15].

Prior studies have mainly been based on emergency department visits and inpatient admissions or mortality data, and very few on general practitioner and primary health-care (PHC) visits [16,17]. The first point of health care contact for patients is through the primary health care system, thus covering most of the diagnosis and treatments for respiratory ailments. The most severe cases are transferred to specialised care for supervised treatment or to an emergency department, if symptoms are more severe. One reason behind the rationale of focusing air pollution research on inpatient and emergency visits has been the availability of data. Hospital registers often cover a large enough population, while primary health care clinics in most places are small units with non-comparable data systems between the units. Due to the lack of PHC data on respiratory symptoms, air pollution effects on respiratory health may thus be underestimated. This was indicated by our previous study, where we observed substantial effects on the number of visits to PHC clinics for asthma [18]. A possible conclusion derived from that study is that low-level exposures may have an effect on less severe types of respiratory health problems. Three studies from Japan [19,20,21] and one from the UK [22] and Chile [23] each used PHC visits data. However, the studies from Japan only used night-time visits, the study from Chile only included patients under 15 years of age, and the study from London, UK, looked at upper respiratory disease only. Studies from Japan and Chile found an association of O_3_ with night-time PHC visits, and a study from London reported a positive association with PM_2.5_ and SO_2_ with upper respiratory diseases.

In Sweden, 80% of the total health care consultations due to respiratory illness are treated at PHC clinics. Since the Swedish health care system is tax-subsidised, PHC data are centrally collected for quality purposes. This gives us an opportunity to investigate the complete effect of air pollution (primarily NO_2_) related to respiratory health, using data on primary health care visits, together with inpatient admissions and emergency department visits. Our study indicated that even at low levels of air pollution there is a significant increased number of PHC visits due to asthma with increasing air pollution [18]. Here, we aim to investigate the impact of air pollution levels on overall respiratory health by studying PHC visits, inpatient, and emergency department visits simultaneously in a multi-municipality setting. This will help us to better understand the association of air pollution with health care utilisation due to respiratory illness in low-level areas.

## 2. Methodology

### 2.1. Study Area

**Figure 1:**
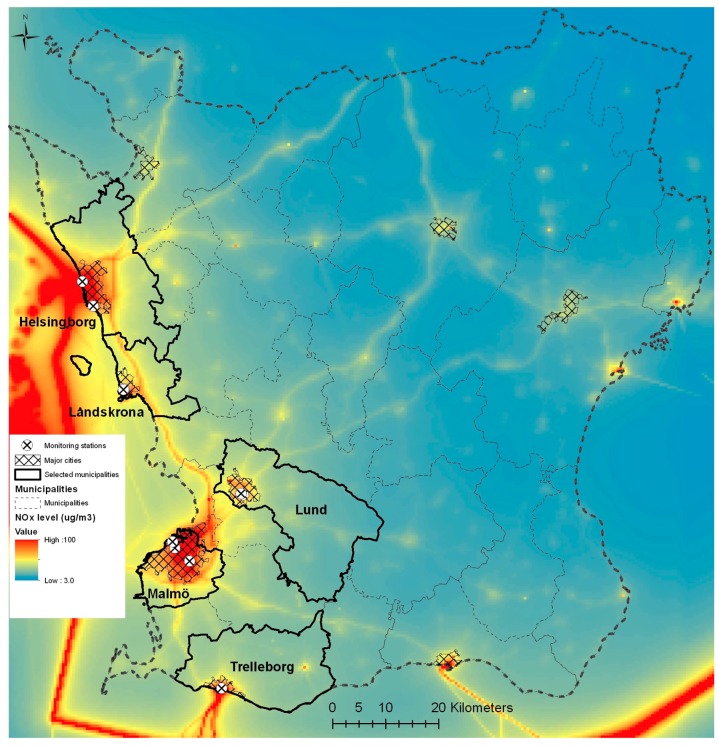
Selected study area and air pollution-monitoring stations.

The study areas were five municipalities in Scania, southern Sweden: Malmö, Helsingborg, Lund, Landskrona, and Trelleborg (Figure 1 shows the Skåne map of air pollution sites and selected municipalities). We selected the municipalities based on population size and availability of monitored data. The air pollution levels for NO_2_, PM_10_, and ozone are presented in Table 1, and the population size for each municipality, together with summary statistics of the health care data, is presented in Table 2. Air pollution concentrations in Scania, relative to other municipalities of Sweden, are rather high, due to rail, ferry, and road links connecting Sweden with the rest of Europe and due to long-distance air pollution from eastern Europe [24,25], although they are well below the WHO and EU air quality guidelines [26,27].

### 2.2. Air Pollution and Meteorological Data

Monitored levels of outdoor air pollution were obtained from the Swedish Environmental Research Institute (IVL) in Sweden. We obtained daily mean values of urban air pollution for PM_10_, O_3_, and NO_2_ for the period from 1 January 2005 to 31 December 2010 for all five municipalities (except for O_3_, which was missing in the Trelleborg municipality). Daily mean values for each municipality were calculated based on all monitoring stations (*N* = 11) within each municipality: three in Malmö, two each in Lund, Helsingborg, and Landskrona, and one in Trelleborg (Figure 1). We had information on each individual’s municipality of residence. Exposure assessments were based on air pollution levels obtained from stations within that municipality of residence (Figure 1). In order to limit exposure misclassification, we only included visits from patients both seeking health care and living in the same municipality. This was done for all three types of visits (primary care, emergency department visits, and hospital admissions). We obtained daily meteorological data for temperature and humidity for the study area from the IVL for stations within the selected municipalities; meteorological data for Trelleborg were missing and information from the Malmö station was used instead [28].

### 2.3. Respiratory Health Care Data

We used the Scania Health Care Register to obtain data on health visits for respiratory outcomes during the study period (2005–2010). The register cover is close to 100% of all cases of individuals seeking health care in the region, due to the reimbursements made to the clinics. Clinics do not receive money from the county council unless they register the patient. Using the International Classification of Diseases, Revision 10 (ICD-10) and the Swedish translation of ICD-10, all health care contacts due to respiratory symptoms by individuals residing in the selected municipalities were identified. ICD-10-J codes and a Swedish translation of the ICD10-J (J00-J99) codes were used to identify respiratory illness.

All respiratory health care visits were categorised into three categories:

- Primary health care visits: All visits to primary health care clinics in the study area, where the physician’s diagnosis was respiratory illness (J-code) and which were categorised as emergency visits, i.e., not pre-booked visits. The number of PHC clinics was 17 in Malmö, six in Lund, eight in Helsingborg, three in Landskrona, and two in Trelleborg. PHC clinics provide health care to both adults and children for non-urgent or non-life-threatening medical problems. PHC clinics also provide preventive health care through vaccination programs and other preventive services. The physicians at PHC clinics typically decide if a patient should be referred to a specialist clinic or if the patient should be treated at the PHC clinic.
- Emergency department visits: Visits to the emergency department at a hospital, where the diagnosis was respiratory illness (J-code).
- Inpatient hospital admission: All hospital admissions, diagnosed as respiratory illness (J-code) by the physician and not pre-planned, were included in the study.

**Figure 2:**
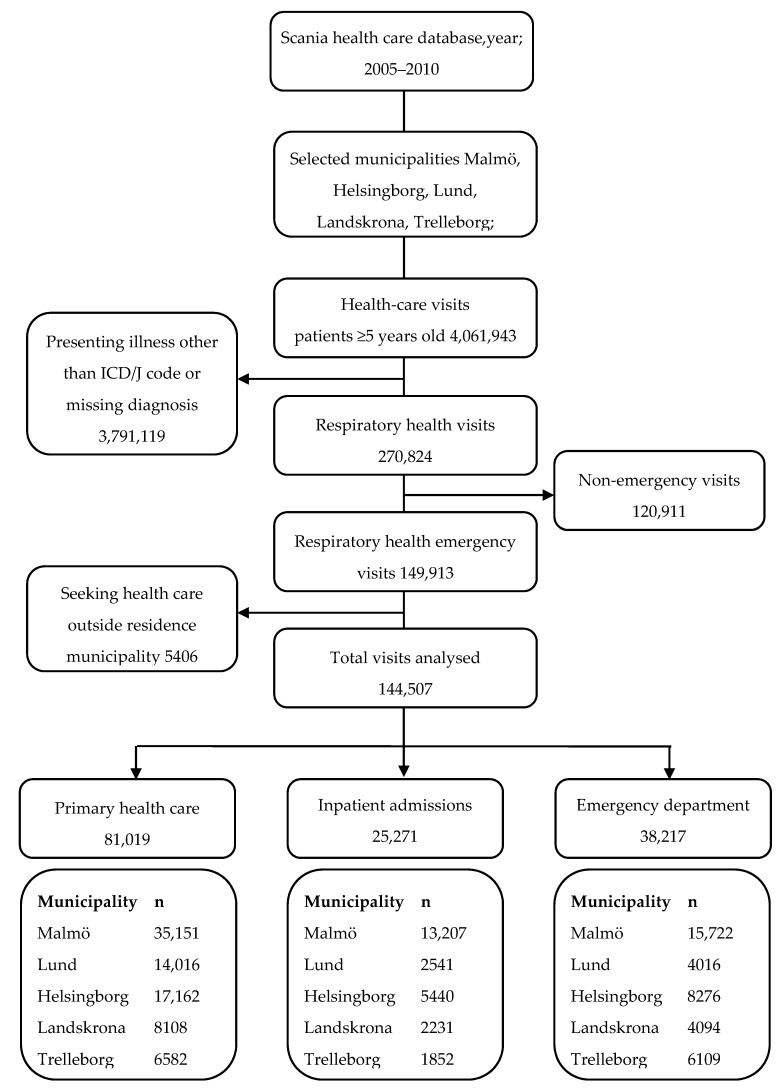
Flow chart showing the selection of health visits for five municipalities in the years 2005–2010.

Figure 2 illustrates the selection of PHC patients, inpatients, and emergency department visits in the study. PHC visits, inpatient admissions, and emergency department visits for respiratory illnesses in the five municipalities were analysed. The visits were restricted to patients above five years of age, since the diagnosis of respiratory health problems at a young age may not be accurate. There were a total of 81,019 PHC visits, 25,271 inpatient admissions, and 38,217 emergency department visits with respiratory illness during the study period.

## 3. Statistical Analysis

### 3.1. Data Analysis

We investigated the hypothesised association between air pollution and respiratory illness, using a case-crossover study design. This design adjusts for seasonality, time trend, and time non-variant factors by design, if the control days are selected suitably [29]. We used calendar month non-overlapping strata, which helps to prevent overlap bias [30], and control days were matched to the day of the week. To further explore if the association between air pollution and respiratory illness may be delayed in time, we combined the case-crossover analyses with a distributed lag non-linear model (DLNM) [31]. We considered the lag cumulative effect over lags of 0–2 days as well as of 0–15 days. Final estimates were presented in percentage increase in health care visits per 10 unit change in pollutant levels.

The statistical model was constructed stepwise; in the first step, the risk was estimated with the main exposure as the only explanatory variable. In the second step, we included smooth functions for temperature and humidity (natural cubic splines with three degrees of freedom each) in the model. In the third step, three pollutants (NO_2_, PM_10_, and O_3_) were added into the same model.

The estimates for the five cities combined were generated by pooling the municipality-specific estimates using fixed effects meta-analyses [32]. All estimates are given as the percentage increase in the number of PHC visits for respiratory symptoms associated with a 10-μg/m^3^ increase in the pollutant level. Statistical analysis was performed using the statistical software R version 2.16 [33] and the statistical package DLNM [1] and MVMETA [32].

### 3.2. Ethics Statement

Our request for primary health care data was granted after formal scrutiny at the Region Skåne’s Health Care Databases.

## 4. Results

The daily mean NO_2_ was highest in Helsingborg (19.2 µg/m^3^, 4 µg/m^3^), higher than the overall average (15.86 µg/m^3^). The daily mean of PM_10_ levels were highest in Landskrona (19.5 µg/m^3^), whereas the daily mean for the whole study area was (17.39 µg/m^3^). The descriptive statistics of air pollution are given in Table 1, and the correlation between air pollutants within and between municipalities is given in the Table S1. Within municipalities, the air pollution concentration correlation was moderate to strong between PM_10_ and NO_2_ (0.54 to 0.16), whereas a negative correlation was observed between O_3_ and NO_2_ (range from −0.37 to −0.55).

### 4.1. Primary Health Care Visits

The number of PHC visits due to respiratory diseases as main illness increased with a 10 µg/m^3^ increase in NO_2_ in all five municipalities in multi-pollutant models, but were only statistically significant in Malmö, with a 1.85% increase (95% CI: 0.52 to 3.20; Table 3 and Figures S1–S3). In Lund, the increase was 1.86% (95% CI: −0.30 to 4.06), but not statistically significant. The number of PHC visits seemed to increase with a 10 µg/m^3^ increase in O_3_ in all municipalities, but was not statistically significant (Table 4 and Figures S1–S3). PM_10_ was not associated with respiratory illness for primary health care visits in the multi-pollutant model (Table 5 and Figures S1–S3). Results for the single-pollutant model are shown in Tables S3–S5. The pooled estimates for the whole study area were generally close to zero (Table S3).

### 4.2. Inpatient Admissions

We did not observe any statistically significant increases in inpatient admissions (Table 3, Table 4 and Table 5, Figures S1–S3, Tables S2–S5).

### 4.3. Emergency Department Visits

An increase of 2.52% (95% CI: 0.44–4.64; Table 5) in Helsingborg for a lag of 0–2 in a multi-pollutant model in emergency department visits was observed with a 10 µg/m^3^ increase for PM_10_, for a lag of 0–2 in a multi-pollutant model (Table 5, Figures S1–S3). For the other municipalities or pollutants, we observed no statistically significant increase. Results for the single-pollutant model are shown in Tables S3–S5. The multi-pollutant pooled estimates for emergency visits were not statistical significant for any pollutants (Tables S2). There were generally no associations observed after a lag of 2 (Figures S1–S3).

## 5. Discussion

Our results suggest an association between air pollution and the number of visits for physician-diagnosed respiratory illness to primary health care on subsequent days, in an area where the air quality is below the WHO guidelines. The increase was 1.85%, (95% CI: 0.52 to 3.20) in Malmö for an average 10 µg/m^3^ increase in NO_2_ the same and two previous days to the visit. In the other three cities, there was no association, and the estimates were close to zero or inconclusive for inpatient and emergency department visits. This was similar to what we had observed earlier [18]. However, this extended study does not give support for effects of air pollution on health care use in the other municipalities or for effects on the number of emergency room visits or inpatient admissions. However, we found a statistically significant increase in emergency room visits of 2.52% (95% CI: 0.44 to 4.64) for a 10 µg/m^3^ increase in PM_10_, which is comparable to results from other studies [34,35,36,37].

It is well known that respiratory health effects are associated with elevations in daily air pollution levels [38,39,40,41]. Our results suggest that the respiratory health impact of air pollution may be underestimated. While previous studies only included emergency department visits [34,35,36,37] or inpatient admissions [42,43,44,45,46], this is the first study where those outcomes are studied together with primary health care visits. The aim was to describe how air pollution is associated with both milder forms of respiratory symptoms (which are mainly handled in primary health care) and more severe forms of respiratory symptoms, namely emergency room visits and inpatient admissions. Important to note is that even milder forms of respiratory symptoms still affect individual quality of life, both physically and economically, due to loss of activity days.

There are several possible explanations for why we observed stronger associations between air pollution and PHC visits than with inpatient admission and emergency department visits. It could be argued that air pollution at the relatively low levels (below the WHO guidelines) observed in the current study would be more likely to result in mild symptoms, which would therefore be treated mainly in PHC centres. Another possible explanation could be that the statistical power to detect associations was higher for the PHC visits than for inpatient admissions or emergency department visits due to the fact that PHC visits are much larger in number than inpatient admissions or emergency department visits. However, the point estimates for the two latter outcomes did not generally indicate any associations, which indicates that statistical power may not be the reason for the lack of associations observed. We restricted the analysis to individuals who were registered residents in the same municipality as they were seeking health care in order to somewhat reduce exposure misclassification. We can, however, not rule out exposure misclassification as an explanation for the lack of association observed for inpatient admissions or emergency department visits. However, we find no good reason to believe that exposure misclassification would differ between the three types of visits.

There was a statistically significant association between NO_2_ and PHC visits in Malmö, and a similar (but not statistically significant association) in Lund, but generally not in the other three municipalities. One possible reason for this lack of finding could be statistical power. Malmö is the largest municipality, but Helsingborg (where no tendency of an association was observed) is larger than Lund, and the concentration range of air pollutants is wider in Helsingborg than in Lund, so the size of the population, or the concentration range alone, do not explain the differences between the cities. It should be noted that the municipalities also differ in terms of population distribution and meteorology, which could influence exposure misclassification and thereby statistical power, which therefore could differ in the five different municipalities. Table S6 describes in detail the population size, the percentage of foreign-born people, gender-stratified mean age, education status, and average monthly income in the selected municipalities during the study period. Malmö was the most populated municipality with the highest number of foreign-born people (30%), Landskrona followed with 24%, while all other municipalities had less than 20%. The education status of the population was significantly different among the different municipalities; Lund, being a university town, had the highest percentage of postsecondary and postgraduate students as compared to other municipalities; however, there was not significant difference in the average monthly salary in the selected municipalities. Sociodemographic difference does effects health-seeking behaviour. Malmö’s having a larger of immigrant population may have resulted in an underutilisation of health care and hence minimise the effect observed in the final analysis. The Swedish health care system is tax funded, with an upper ceiling of SEK 1100 (around 150 US$) for hospital health care and of SEK 2000 (around 300 US$) for dispensed medications. For children, every kind of health care is completely free of charge. For to this reason, it is reasonable to believe that economic status will have little or no effect on the health-seeking behaviour among the selected municipalities.

Various biological mechanisms have been identified explaining the effects of pollutants on our respiratory system. These processes include activation of innate immune responses [47], impact on elements of IgE-mediated immunity [48], resulting in asthmatic symptoms, or induction of oxidative stress, resulting in COPD and asthma. Particulate matter moderated by pro-inflammatory effects due to oxidative response with the metal content of particles results in injury to the air way lining [1]. O_3_ may mediate by effecting a nociceptive response (altering pain response), resulting in impaired air passage clearances, leading to bronchial obstruction [49] and increasing neutrophilic inflammation, increasing the influx of dendritic cells and macrophages, which results in increased airway permeability and bronchial reactivity [50]. NO_2_ exposure also may result in an influx of polymorphonuclear leukocytes (PMNs), resulting in increased bronchial activity [51].

A major strength of our study is that we were able to study three types of health care visits in the same study, namely primary health care visits, emergency department visits, and inpatient admissions. However, comparison of the three types of visits was not always straight-forward. Although it is reasonable to assume that primary health care visits represent milder symptoms than emergency department visits or inpatient visits, there are certainly exceptions from when this assumption is true, which is a limitation of the study. A major strength of the study is that we could study the association between air pollution and respiratory health visits in five separate municipalities, where at least one individual air pollution station was located close to the most populated section of the municipality. If we had not been able to distinguish between different municipalities, but had only investigated all municipalities together, the conclusion of the study would have been different, given that the pooled estimates were all close to zero. This could indicate that in order to study daily variations in air pollution levels in association with respiratory health in low-level areas, it is important to have a high spatial resolution. Another major strength is that we had access to data records from the regional body of health care in our region. Our results are therefore not prone to selection bias, and it is reasonable to believe that records are independent of air pollution levels, so the information bias should also be minimal.

Interestingly, the strongest associations was observed for NO_2_, and not for PM_10_, as in many other studies, where it was shown that an increase in PM_10_ levels results in a significant increase in emergency care visits [52,53,54,55] and hospital admissions due to respiratory illness [56,57,58,59]. A possible reason for this lack of association could be the very low levels of PM_10_ throughout the study period compared to the studies above. NO_2_ is in our setting a strong marker for combustion-related air pollution, mainly traffic, which is the largest source of local emissions of air pollution in Scania.

## 6. Conclusions

The results suggest that short-term elevations in NO_2_ may increase the number of visits to primary health care clinics for respiratory symptoms, but not the emergency department visits or inpatient admissions. The results were only statistical significant in the largest municipality (Malmö), and the pooled results were close to zero for all pollutants and types of health care. This could indicate that in order to study daily variations in air pollution levels in association with respiratory health in low-level areas, it is of importance to have a high spatial resolution. Short-term elevations in NO_2_ may have had an effect on respiratory symptoms in the study area, but the effect was not observable in most municipalities studied in the present study. The inconclusive results may be due to the lack of statistical power, which in turn may be due to the size of the study area or to the generally low levels of air pollution in the study area.

## Figures and Tables

**Table 1:** Descriptive statistics of air pollution data during the study period (2005–2010).

	PM_10_ µg/m^3^				NO_2_ µg/m^3^				O_3_ µg/m^3^			
	Min	Median	Mean (SD)	Max	Min	Median	Mean (SD)	Max	Min	Median	Mean (SD)	Max
**Malmö**	2.7	14.7	16.4 (8.0)	60.9	3.4	16.4	17.8 (7.8)	53.3	4.3	54.7	53.3 (17.3)	138
**Lund**	0.1	12.4	14.4 (9.7)	157	0.4	11.5	13.2 (7.8)	51.5	5.4	56.7	56.3 (7.7)	123.7
**Helsingborg**	0.1	15.7	17.8 (11.1)	114	2.0	17.4	19.2 (9.6)	62.5	6.9	56.5	56.2 (15.8)	138
**Landskrona**	2.0	17.3	19.5 (11.0)	75.6	0.2	12.5	13.9 (7.1)	54.5	5.1	58.2	57.4 (15.0)	108.7
**Trelleborg**	0.8	16.7	18.4 (9.6)	92.2	2.3	15.9	16.9 (8.2)	64.7	-	-	-	-

SD: Standard Deviation.

**Table 2:** Population size and descriptive statistics of primary health care (PHC) visits, inpatient admissions, and emergency department visits in the five municipalities during the study period (2005–2010).

Municipality	Population Size	PHC Visits	Inpatient Admissions	ED Visits
	*N*	Daily Mean (SD)	Daily Mean (SD)	Daily Mean (SD)
Malmö	298,963	16.0 (9.1)	6.0 (3.0)	7.2 (3.5)
Lund	110,488	6.8 (4.3)	1.2 (1.1)	2.9 (1.9)
Helsingborg	129,177	8.1 (5.0)	2.8 (1.6)	3.8 (2.2)
Landskrona	41,724	4.0 (2.4)	1.0 (1.0)	2.1 (1.6)
Trelleborg	42,219	4.2 (2.5)	0.9 (1.0)	3.0 (2.0)

**Table 3:** Percent change in the number of visits to PHC clinics, inpatient admissions, and emergency department visits with 95% CI associated with a 10 µg/m^3^ increase in NO_2_ lag 0–2 in five municipalities during 2005–2010 in multi-pollutant models.

Municipality	PHC % Change (95% CI)	Inpatient % Change (95% CI)	Emergency Department % Change (95% CI)
Malmö	1.85 (0.52 to 3.20)	0.72 (−1.10 to 2.58)	0.88 (−0.77 to 2.57)
Lund	1.86 (−0.30 to 4.06)	0.64 (−4.25 to 5.82)	1.19 (−2.84 to 5.42)
Helsingborg	0.09 (−1.33 to 1.54)	0.91 (−1.08 to 2.94)	−1.09 (−2.87 to 0.73)
Landskrona	−3.14 (−7.68 to 1.43)	−2.10 (−10.74 to 7.51)	−0.02 (−5.91 to 6.29)
Trelleborg	0.83 (−1.77 to 3.51)	0.53 (−2.21 to 3.35)	2.86 (−7.79 to 14.93)

**Table 4:** Percent change in the number of visits to PHC clinics, inpatient admissions, and emergency department visits with 95% CIs associated with a 10 µg/m^3^ increase in O_3_ lag 0–2 in five municipalities during 2005–2010 in multi-pollutant models.

Municipality	PHC % Change (95% CI)	Inpatient % Change (95% CI)	Emergency Department % Change (95% CI)
Malmö	0.21 (−0.53 to 0.95)	0.91 (−0.11 to 1.94)	0.75 (−0.19 to 1.71)
Lund	0.73 (−0.42 to 1.89)	1.57 (−1.14 to 4.37)	2.07 (−0.16 to 4.36)
Helsingborg	0.79 (−0.25 to 1.84)	−0.54 (−1.97 to 0.91)	0.20 (−1.13 to 1.54)
Landskrona	1.70 (−1.48 to 5.01)	1.37 (−4.79 to 8.01)	−0.16 (−4.09 to 3.98)
Trelleborg *	-	-	-

* In Trelleborg, O_3_ data were missing.

**Table 5:** Percent change in the number of visits to PHC clinics, inpatient admissions, and emergency department visits with 95% CIs associated with a 10 µg/m^3^ increase in PM_10_ lag 0–2 in five municipalities during 2005–2010 in multi-pollutant models.

Municipality	PHC % Change (95% CI)	Inpatient % Change (95% CI)	Emergency Department % Change (95% CI)
Malmö	0.29 (−0.72 to 1.31)	0.41 (−0.98 to 1.83)	0.10 (−1.16 to 1.39)
Lund	−0.67 (−2.66 to 1.37)	−0.05 (−4.68 to 4.83)	0.25 (−3.68 to 4.37)
Helsingborg	0.38 (−1.20 to 1.99)	0.59 (−1.61 to 2.84)	2.52 (0.44 to 4.64)
Landskrona	1.45 (−2.70 to 5.81)	0.10 (−8.33 to 9.40)	−2.21 (−7.81 to 3.77)
Trelleborg	1.07 (−1.13 to 3.32)	0.46 (−1.94 to 2.93)	−0.19 (−2.18 to 1.85)

## Supplementary Materials

The following are available online at www.mdpi.com/1660-4601/14/6/587/s1, Table S1: Spearman correlation coefficients between air pollutants within and between the five municipalities in Scania, Sweden, 2005–2010, Table S2: Pooled estimates of percent change in the number of visits to Primary Health Care Clinics (PHCs), Inpatient admissions, and Emergency Department visits with 95% Confidence Intervals (CIs) associated with a 10 μg/m^3^ increase in NO_2_, O_3_, and PM_10_ lag 0–2 in five municipalities during 2005–2010, Table S3: Percent change in the number of visits to Primary Health Care Clinics (PHCs), Inpatient admissions, and Emergency Department visits with 95% Confidence Intervals (CIs) associated with a 10 μg/m^3^ increase in PM_10_ single-pollutant model over lag 0–2 in five municipalities during 2005–2010, Table S4: Percent change in the number of visits to Primary Health Care Clinics (PHCs), Inpatient admissions, and Emergency Department visits with 95% Confidence Intervals (CIs) associated with a 10 μg/m^3^ increase in O_3_ single-pollutant model over lag 0–2 in five municipalities during 2005–2010, Table S5: Percent change in the number of visits to Primary Health Care Clinics (PHCs), Inpatient admissions, and Emergency Department visits with 95% Confidence Intervals (CIs) associated with a 10 μg/m^3^ increase in NO_2_ single-pollutant model over lag 0–2 in five municipalities during 2005–2010, Table S6: Selected Municipalities social demographic characteristics for the year 2010, Table S7: Weather Condition in Malmö Municipality from year 2005 till 2010, Figure S1: Lag response curve for 10 μg/m^3^ s increase in NO_2_ level with Respiratory health care visits (multi-pollutant models), Figure S2: Lag response curve for 10 μg/m^3^ s increase in O_3_ level with Respiratory health care visits (multi-pollutant models), Figure S3: Lag response curve for 10 μg/m^3^ s increase in PM_10_ level with Respiratory health care visits (multi-pollutant models).
