# Supplementary material for: Short-Term Associations between Air Pollution Concentrations and Respiratory Health—Comparing Primary Health Care Visits, Hospital Admissions, and Emergency Department Visits in a Multi-Municipality Study
Source: Int J Environ Res Public Health. 2017 May 31;14(6):587. doi: 10.3390/ijerph14060587 (PMC5486273; doi:10.3390/ijerph14060587)
Supplement: Supplementary file 1 [file ijerph-14-00587-s001.pdf]

# Short-Term Associations between Air Pollution Concentrations and Respiratory Health—Comparing Primary Health Care Visits, Hospital Admissions, and Emergency Department Visits in a Multi-Municipality Study

**Tahir Taj [1,*], Ebba Malmqvist [1], Emilie Stroh [1], Daniel Oudin Åström [2,3], Kristina Jakobsson [1] and Anna Oudin [1,3]**

[1] Department of Occupational and Environmental Medicine, Lund University, SE-22100 Lund, Sweden; ebba.malmqvist@med.lu.se (E.M.); emilie.stroh@med.lu.se (E.S.); kristina.jakobsson@med.lu.se (K.J.); anna.oudin@umu.se (A.O.)
[2] Center for Primary Health Care Research, Department of Clinical Science, Malmö, Lund University, SE-205 02 Malmö, Sweden; daniel.oudin_astrom@med.lu.se
[3] Department of Occupational and Environmental Medicine, Umeå University, Umeå, SE-22100 Lund, Sweden
* Correspondence: tahir.taj@med.lu.se

Academic Editor: tahir.taj@med.lu.se
Received: 22 March 2017; Accepted: 27 May 2017; Published: date

**Corresponding author:**

Tahir Taj
Lund University Laboratory Medicine
Division of Occupational and Environmental Medicine
Lund, Sweden
Telephone: +46 72 588 4202
E-mail: tahir.taj@med.lu.se

Acknowledgements

The authors would like to thank FORMAS, The Swedish Research Council for Environment, Agricultural Sciences and Spatial Planning and the Faculty of Medicine, Lund University, Sweden for funding the research project.

**Supplementary Material, Table 1:** Spearman correlation coefficients between air pollutants within and between the five municipalities in Scania, Sweden, 2005-2010

| **Municipality** | | **Malmö** | | | **Lund** | | | **Helsingborg** | | | **Landskrona** | | | **Trelleborg** | |
|---|---|---|---|---|---|---|---|---|---|---|---|---|---|---|---|
| | | **$O_3$** | **$NO_2$** | **$PM_{10}$** | **$O_3$** | **$NO_2$** | **$PM_{10}$** | **$O_3$** | **$NO_2$** | **$PM_{10}$** | **$O_3$** | **$NO_2$** | **$PM_{10}$** | **$NO_2$** | **$PM_{10}$** |
| **Malmö** | **$O_3$** | 1 | | | | | | | | | | | | | |
| | **$NO_2$** | -0.53 | 1 | | | | | | | | | | | | |
| | **$PM_{10}$** | 0.06 | 0.26 | 1 | | | | | | | | | | | |
| **Lund** | **$O_3$** | 0.84 | -0.43 | 0.06 | 1 | | | | | | | | | | |
| | **$NO_2$** | -0.35 | 0.65 | 0.19 | -0.54 | 1 | | | | | | | | | |
| | **$PM_{10}$** | 0.17 | 0.16 | 0.74 | 0.04 | 0.19 | 1 | | | | | | | | |
| **Helsingborg** | **$O_3$** | 0.84 | -0.24 | 0.01 | 0.84 | 0.16 | 0.02 | 1 | | | | | | | |
| | **$NO_2$** | -0.37 | 0.65 | 0.31 | -0.44 | 0.24 | 0.33 | -0.37 | 1 | | | | | | |
| | **$PM_{10}$** | 0.07 | 0.33 | 0.59 | 0.06 | 0.42 | 0.63 | 0.00 | 0.37 | 1 | | | | | |
| **Landskrona** | **$O_3$** | 0.91 | -0.04 | 0.02 | 0.90 | 0.00 | -0.02 | 0.93 | -0.11 | -0.10 | 1 | | | | |
| | **$NO_2$** | -0.49 | 0.08 | 0.14 | -0.63 | 0.12 | 0.26 | -0.46 | 0.24 | 0.35 | -0.55 | 1 | | | |
| | **$PM_{10}$** | -0.09 | 0.54 | 0.69 | -0.05 | 0.49 | 0.84 | -0.06 | 0.36 | 0.62 | -0.10 | 0.41 | 1 | | |
| **Trelleborg** | **$NO_2$** | --- | 0.30 | 0.22 | --- | 0.48 | 0.30 | --- | 0.43 | 0.25 | --- | 0.44 | 0.44 | 1 | |
| | **$PM_{10}$** | --- | 0.18 | 0.61 | --- | 0.20 | 0.67 | --- | 0.23 | 0.48 | --- | 0.28 | 0.80 | 0.40 | 1 |

**Supplementary Material, Table 2:** Pooled estimates of percent change in the number of visits to Primary Health Care Clinics (PHCs), Inpatient admissions, and Emergency Department visits with 95% Confidence Intervals (CIs) associated with a 10 µg/m3 increase in $NO_2$, $O_3$, and $PM_{10}$ lag 0-2 in five municipalities during 2005–2010.

| | **PHC % Change (95% CI)** | **Inpatient % Change (95%** | **Emergency Department** |
|---|---|---|---|
| **$NO_2$** | -1.09 (-3.81 to 1.69) | -3.08 (-6.07 to 0.01) | -0.31 (-2.47 to 1.91) |
| **$O_3$** | 0.28 (-0.20 to 0.76) | 0.29 (-0.19 to 0.77) | -0.05 (-0.43 to 0.33) |
| **$PM_{10}$** | 0.25 (-0.61 to 1.13) | -0.17 (-2.08 to 1.77) | -0.10 (-1.17 to 0.99) |

**Supplementary Material, Table 3:** Percent change in the number of visits to Primary Health Care Clinics (PHCs), Inpatient admissions, and Emergency Department visits with 95% Confidence Intervals (CIs) associated with a 10 µg/m3 increase in $PM_{10}$ single-pollutant model over lag 0-2 in five municipalities during 2005–2010.

| Municipality | PHC<br>% Change (95% CI) | Inpatient<br>% Change (95% CI) | Emergency Department<br>% Change (95% CI) |
|---|---|---|---|
| **Malmö** | 0.36 (-0.62 to 1.34) | 0.08 (-1.27 to 1.45) | -0.04 (-1.26 to 1.21) |
| **Lund** | 0.39 (-1.02 to 1.83) | -1.63 (-4.93 to 1.81) | 1.13 (-1.84 to 4.21) |
| **Helsingborg** | 1.05 (0.01 to 2.11) | -0.22 (-1.69 to1.28 ) | 0.76 (-0.58 to 2.12) |
| **Landskrona** | 0.09 (-1.56 to 1.76) | -2.01 (-5.30 to 1.42) | -0.44 (-2.61 to 1.79) |
| **Trelleborg** | 1.95 (0.10 to 3.84) | -0.21 (-4.03 to 3.78) | -1.11 (-3.23 to 1.07) |

**Supplementary Material, Table 4:** Percent change in the number of visits to Primary Health Care Clinics (PHCs), Inpatient admissions, and Emergency Department visits with 95% Confidence Intervals (CIs) associated with a 10 µg/m3 increase in $O_3$ single-pollutant model over lag 0-2 in five municipalities during 2005–2010.

| **Municipality** | **PHC % Change (95% CI)** | **Inpatient % Change (95% CI)** | **Emergency Department % Change (95% CI)** |
|---|---|---|---|
| **Malmö** | -0.08 (-0.72 to 0.56) | 0.60 (-0.03 to 1.51) | 0.63 (-0.19 to 1.47) |
| **Lund** | -0.40 (-1.36 to 0.58) | 1.39 (-0.80 to 3.63) | 0.31 (-1.46 to 2.10) |
| **Helsingborg** | 0.19 (-0.68 to 1.06) | 0.91 (-0.30 to 2.14) | 0.47 (-0.64 to 1.60) |
| **Landskrona** | -0.10 (-1.51 to 1.34) | 2.51 (-0.32 to 5.42) | 1.35 (-0.60 to 3.35) |
| **Trelleborg*** | --- | --- | --- |

*Trelleborg $O_3$ data was missing

**Supplementary Material, Table 5:** Percent change in the number of visits to Primary Health Care Clinics (PHCs), Inpatient admissions, and Emergency Department visits with 95% Confidence Intervals (CIs) associated with a 10 µg/m3 increase in $NO_2$ single-pollutant model over lag 0-2 in five municipalities during 2005–2010.

| Municipality | PHC % Change (95% CI) | Inpatient % Change (95% CI) | Emergency Department % Change (95% CI) |
|---|---|---|---|
| **Malmö** | 1.26 (0.15 to 2.39) | 0.21 (-1.34 to 1.78) | 0.23 (-1.18 to 1.67) |
| **Lund** | 1.77 (0.21 to 3.35) | -2.24 (-5.61 to 1.26) | -0.19 (-3.01 to 2.71) |
| **Helsingborg** | 0.56 (-0.53 to 1.67) | -1.29 (-2.79 to 0.25) | -0.77 (-2.16 to 0.64) |
| **Landskrona** | -0.83 (-2.81 to 1.18) | -4.12 (-7.92 to -0.14) | -1.06 (-3.78 to 1.75) |
| **Trelleborg** | 0.93 (-1.40 to 3.31) | -0.99 (-5.39 to 3.64) | -0.53 (-3.09 to 2.12) |

**Supplement Material Table 6:** Selected Municipalities social demographic characteristics for the year 2010.

| | | Population | Age | | Education | | | | Income |
|---|---|---|---|---|---|---|---|---|---|
| **Municipalities** | | **Total** | **Mean (Yrs)** | **Foreign born** | **Primary*** | **Secondary**** | **Post-Secondary†** | **Post-graduate††** | **SEK ^** |
| **Malmö** | Men | 1 46 750 | 37.8 | 4 5328 (31%) | 21 719 (21%) | 43 670 (41%) | 38 671 (37%) | 1 382 (1%) | 25 100 |
| | Women | 1 52 213 | 40.2 | 4 5021 (30%) | 21 126 (20%) | 40 295 (38%) | 44 653 (42%) | 786 (1%) | 24 000 |
| | Total | 2 98 963 | 39.0 | 90 349 (30%) | 42 845 (20%) | 83 965 (40%) | 83 324 (40%) | 2 168 (1%) | 24 300 |
| **Lund** | Men | 55 084 | 36.7 | 9 289 (17%) | 5 282 (12%) | 11 440 (28%) | 21 790 (52%) | 3 006 (7%) | 24 900 |
| | Women | 55 404 | 39.4 | 9 940 (18%) | 4 554 (11%) | 10 650 (26%) | 24 267 (59%) | 1 647 (4%) | 23 900 |
| | Total | 1 10 488 | 38.1 | 19 229 (17%) | 9 836 (12%) | 22 090 (27%) | 46 057 (56%) | 4 653 (6%) | 24 200 |
| **Landskrona** | Men | 20 769 | 40.2 | 5 013 (24%) | 3 839 (26%) | 7 770 (52%) | 3 131 (21%) | 78 (0.5%) | 24 900 |
| | Women | 20 955 | 42.5 | 5 078 (24%) | 3 637 (25%) | 6 766 (47%) | 4 084 (28%) | 52 (0,4%) | 23 500 |
| | Total | 41 724 | 41.4 | 10 091 ()24% | 7 476 (26%) | 14 536 (50%) | 7 215 (25%) | 130 (0.4%) | 23 800 |
| **Helsingborg** | Men | 63 271 | 39.7 | 12 236 (19%) | 11 027 (24%) | 21 589 (47%) | 13 223 (29%) | 345 (0.7%) | 26 700 |
| | Women | 65 906 | 42.3 | 13 017 (29%) | 10 015 (21%) | 20 454 (44%) | 16 346 (35%) | 164 (0.3%) | 24 600 |
| | Total | 1 29 177 | 41.0 | 25 253 (20%) | 21 042 (23%) | 42 043 (45%) | 29 569 (31%) | 509 (0.5%) | 25 100 |
| **Trelleborg** | Men | 21 049 | 40.9 | 3 027 (14%) | 4 190 (28%) | 7 899 (52%) | 2 970 (20%) | 49 (0.3%) | 26 300 |
| | Women | 21 170 | 42.9 | 2 973 ()14% | 3 453 (23%) | 7 365 (49%) | 4 122 (28%) | 38 (0.3%) | 24 000 |
| | Total | 42 219 | 41.9 | 6 000 (14%) | 7 643 (25) | 15 264 (51%) | 7 092 (24%) | 87 (0.3%) | 24 500 |

* Primary and secondary education up to 10 years

**Upper secondary education, 2 years or less

†Upper secondary education 3 years

††Post-secondary education, less than 3 years

^^Average monthly salary in Swedish Krona 1 USD ≈ 9 SEK

Data obtained from Statistics Sweden [1]

**Supplement Table 7 Weather Condition in Malmö Municipality from year 2005 till 2010**

| | Min | 1st quantile | Median | Mean (Sd) | 3rd quantile | Max |
|---|---|---|---|---|---|---|
| Temperature °C | 9.8 | 4.1 | 9.4 | 9.5 (7.1) | 15.5 | 26.4 |
| Humidity | 37.4 | 67.3 | 76.2 | 74.6 (11.3) | 83.2 | 95.9 |
| Rain Fall | 0.00 | 0.00 | 0.00 | 1.8 (4.4) | 1.6 | 78.5 |

**Supplementary Material, Figure 1:** Lag response curve for 10 µg/m3 s increase in $NO_2$ level with Respiratory health care visits (multi-pollutant models).

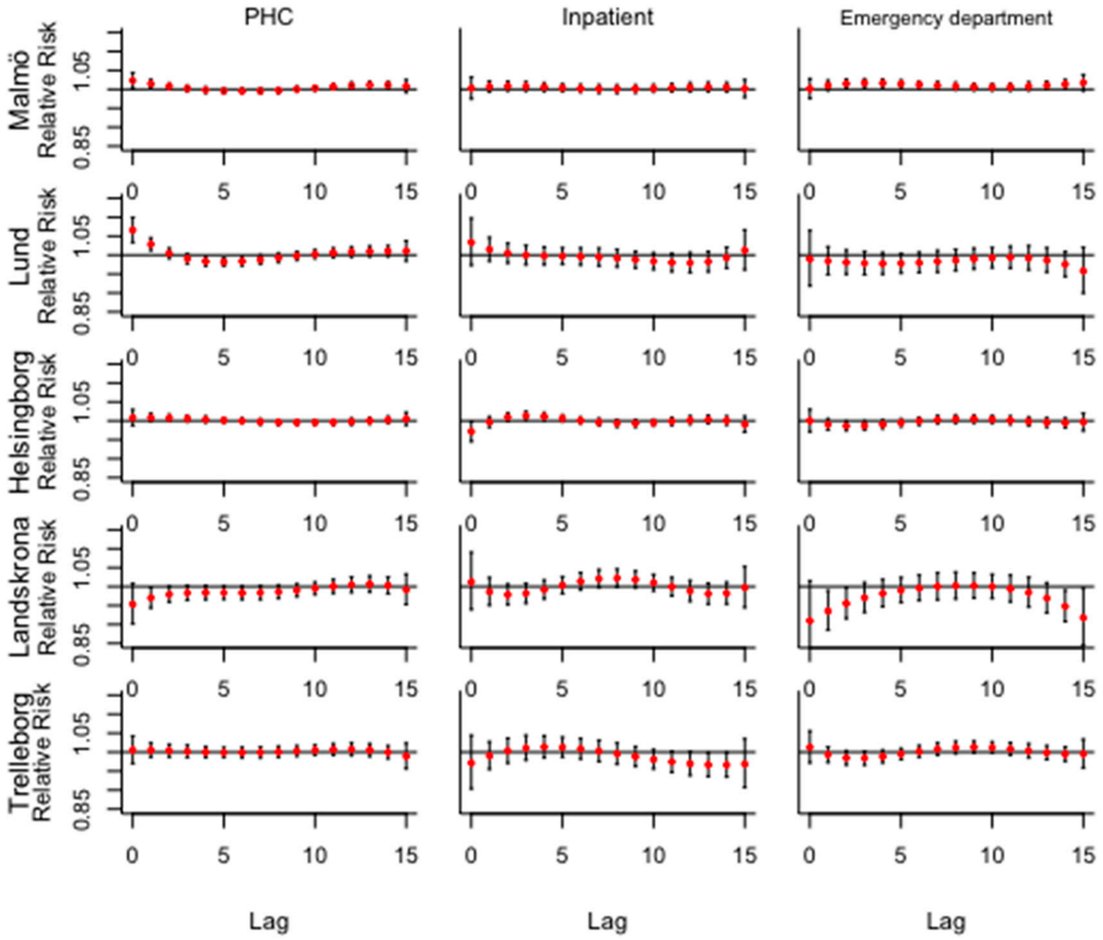

**Supplementary Material, Figure 2:** Lag response curve for 10 µg/m3 s increase in $O_3$ level with Respiratory health care visits (multi-pollutant models).

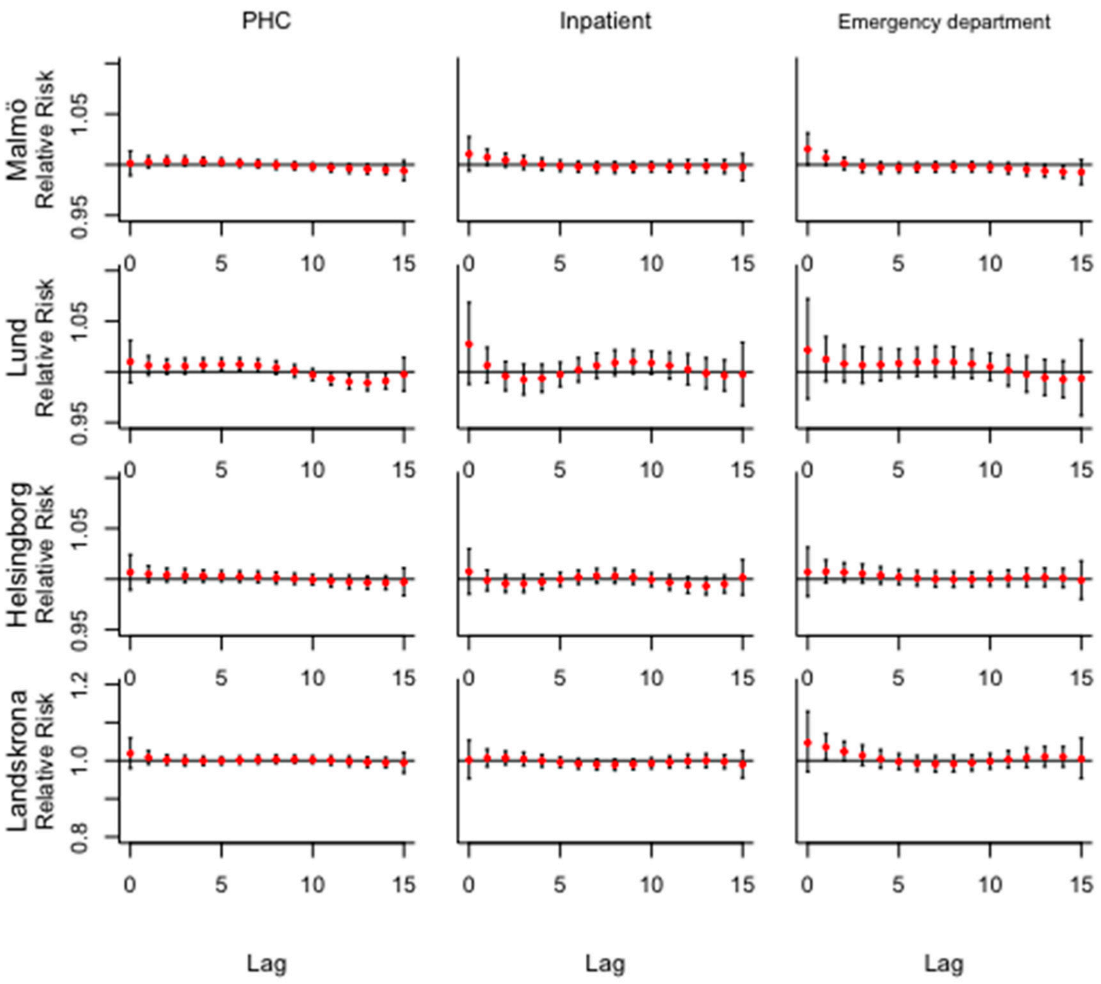

**Supplementary Material, Figure 3:** Lag response curve for 10 µg/m3 s increase in $PM_{10}$ level with Respiratory health care visits (multi-pollutant models).

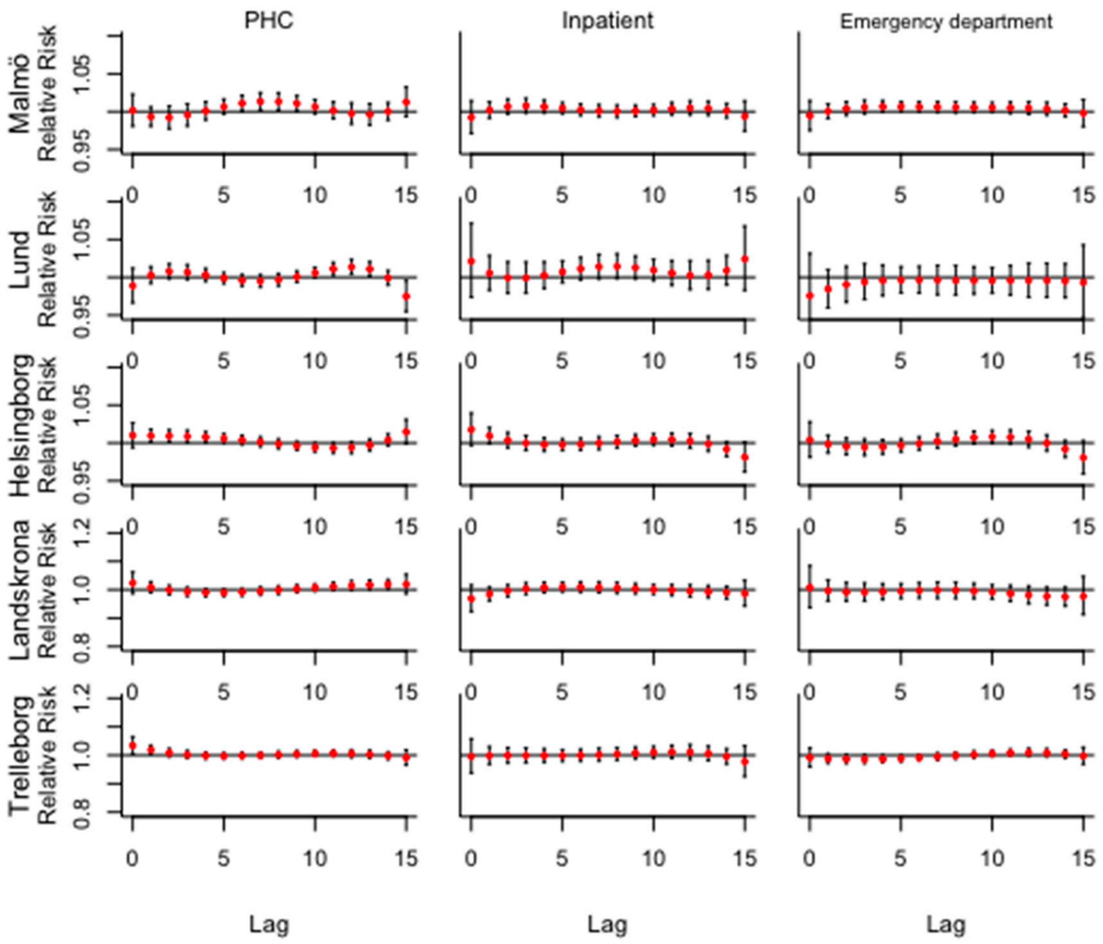

## Reference:

1. Sweden, S. *Statistical database*. 2017  12 May 2017]; Available from: http://www.scb.se/en/.
